# Physical activity and blood gene expression profiles: the Norwegian Women and Cancer (NOWAC) Post-genome cohort

**DOI:** 10.1186/s13104-020-05121-2

**Published:** 2020-06-11

**Authors:** Karina Standahl Olsen, Marko Lukic, Kristin Benjaminsen Borch

**Affiliations:** Department of Community Medicine, UiT The Arctic University of Norway, Tromsö, Norway

**Keywords:** Physical activity, Transcriptomics, Blood, Immunology

## Abstract

**Objectives:**

The influence of physical activity (PA) on the immune system has emerged as a new field of research. Regular PA may promote an anti-inflammatory state in the body, thus contributing to the down-regulation of pro-inflammatory processes related to the onset and progression of multiple diseases. We aimed to assess whether overall PA levels were associated with differences in blood gene expression profiles, in a cohort of middle-aged Norwegian women. We used information from 977 women included in the Norwegian Women and Cancer (NOWAC) Post-genome cohort. Information on PA and covariates was extracted from the NOWAC database. Blood samples were collected using the PAXgene Blood RNA collection system, and gene expression profiles were measured using Illumina microarrays. The R-package limma was used for the single-gene level analysis. For a target gene set analysis, we used the global test R-package with 48 gene sets, manually curated from the literature and relevant molecular databases.

**Results:**

We found no associations between overall PA levels and gene expression profiles at the single-gene level. Similarly, no gene sets reached statistical significance at adjusted p < 0.05. In our analysis of healthy, middle-aged Norwegian women, self-reported overall PA was not associated with differences in blood gene expression profiles.

## Introduction

Physical activity (PA) is one of the major modifiable risk factors for several diseases, along with other lifestyle factors such as smoking, alcohol consumption, and diet. PA is a complex phenomenon, which includes concepts such as exercise and training, as well as occupational, leisure time, household and transportation activities, all at different intensity, duration and frequency [1]. Studies have shown that PA is associated with reduced risk of both communicable [2] and non-communicable diseases like cardiovascular diseases, diabetes, overweight/obesity, and cancers of the breast, endometrium and colon [3–7]. From a public health perspective, the health effects of the total PA level of the general population is of particular interest.

The physiological and molecular mechanisms of the association between PA and health are not fully understood. At the physiological level, PA influences energy expenditure, metabolism, cardiorespiratory and muscular fitness, and body composition, with subsequent consequences for disease risk [1]. The main hormonal systems at play in the link between PA and disease include sex steroids [8, 9], adipokines [10], as well as insulin and insulin-like growth factors [11]. In recent years, the influence of PA on the immune system has emerged as a new field of research. Regular PA may promote an anti-inflammatory state in the body, thus contributing to the down-regulation of pro-inflammatory processes related to the onset and progression of multiple diseases [12]. Exercise in acute bouts in clinical trials in humans, on the other hand, may lead to muscle tissue damage and localized inflammation, with systemic release of cytokines that may act either pro- or anti-inflammatory [12, 13]. Hence, the immunological response to PA differs according to the intensity and duration of PA, but several other factors also modify the response. These factors include age, diet, level of PA, and baseline level of inflammation in the body [12, 14].

New epidemiological evidence is demonstrating the importance of increased everyday PA and reduced sedentary behavior to minimize the risk of disease, and new insights on immunological mechanisms of exercise are emerging. However, understanding how PA levels in a general population influences immunological mechanisms remains a challenge. Here, we have assessed whether PA levels are associated with differences in the gene expression profiles of immune cells in whole-blood samples collected in the NOWAC Post-genome cohort, a nationally representative, population-based cohort of middle-aged women.

## Main text

### Methods

The NOWAC study [15] is a nationally representative, prospective cohort study which includes more than 170,000 middle-aged women. The participants answered one or more 4- or 8-page questionnaires on lifestyle, dietary factors and health. In the years 2003–2006, approx. 50,000 of the NOWAC women donated a blood sample eligible for gene expression analysis and answered concurrently a 2-page questionnaire, collectively forming the NOWAC Post-genome cohort (further details in [16]). For the present cross-sectional study, 977 cancer-free women were randomly drawn from the NOWAC Post-genome cohort.

Information on overall PA and age at inclusion was extracted from questionnaires answered no more than 1 year prior to blood sampling. All other variables were extracted from the 2-page questionnaire accompanying the blood sample. PA level was self-reported on a 10-increment scale from 1 to 10. The question on PA was stated as follows (see Additional file 1: Table S1): “*By physical activity we mean both work in and outside the home, as well as training/exercise and other physical activity, such as walking,* etc*. Mark the number that best describes your level of physical activity*.” For the present analyses, we defined five PA categories by combining levels 1 + 2 (very low), 3 + 4 (low), 5 + 6 (moderate), 7 + 8 (high), and 9 + 10 (very high) from the 10-increment scale. Smoking (during the last week yes/no) and use of medication (during the last week yes/no) were defined as smoking or taking medication during the past week before giving the blood sample. Medication was used as a proxy to gain a comprehensive impression of the participants’ health status, and was grouped according to the Anatomical Therapeutic Chemical (ATC) classification, and the following classes were assessed: N (nervous system, including analgesics), C (cardiovascular system), M (musculo-skeletal system, including non-steroidal anti-inflammatory drugs), and B (blood and blood forming organs). We excluded the lowest PA category from analyses due to low n (36), as well as high body mass index (BMI) (27.8) and high frequency of medication use (78%) in that group. We also excluded women with missing information on either PA, BMI, smoking, or medication use (n = 70).

Blood samples were collected using the PAXgene Blood RNA collection system, Preanalytix/Qiagen, Hilden, Germany), they were kept at − 80 °C until shipment to the Genomics Core Facility at the Norwegian University of Science and Technology for analysis. Total RNA was isolated in accordance with the manufacturer’s protocol (PAXgene Blood miRNA isolation Kit). RNA purity was assessed by NanoDrop ND 8000 spectrophotometer (ThermoFisher Scientific, Wilmington, DE, USA), and RNA integrity by Bioanalyzer capillary electrophoresis (Agilent Technologies, Palo Alto, CA, USA). The mRNA was amplified and labeled using the Illumina TotalPrepT-96 RNA Amplification Kit (Ambion Inc., Austin, TX, USA), and hybridized to Illumina HumanHT-12 Expression BeadChip microarrays (Illumina, Inc. San Diego, CA, USA). The raw microarray images were processed in Illumina GenomeStudio. Preprocessing of the microarray dataset is described in [17]. The main steps of the preprocessing included (1) removal of outliers, (2) background correction (using the R package limma, function nec), and (3) probe filtering based on Illumina quality control measures, detection in < 1% of samples, or probes that were not mapped to an Illumina ID. The dataset was quantile normalized and log2 transformed using the R package lumi, functions lumiN and lumiT. The R packages lumi: nuID2RefSeqID and illuminaHumanv4.db were used for annotation of Illumina IDs to gene symbols. The final gene expression dataset included 7741 probes and 977 individuals. After applying the exclusion criteria based on PA levels and missing information on covariates, our analytical sample size consisted of 871 women.

We used independent sample t-test and chi-square statistics to compare different levels of physical activity according to age, smoking, BMI, and use of medication at the time of the blood sample. We looked for differential expression first at the single gene level (R package limma [18]), and secondly at the gene set level (R package: global test [19]). In the search for differentially expressed single genes, we compared PA levels low versus high, and low versus very high, using false discovery rate-adjusted p < 0.05 as a significance threshold. Secondly, we checked for linear trends in gene expression, using the four highest PA categories as a continuous variable. All single gene level analyses were adjusted for variables that were significantly different (p < 0.05) between one or more comparison groups: BMI, smoking, and use of ATC class N medications. In addition to single-gene level analyses, we carried out a targeted gene set level analysis using global test [19]. As input, we curated gene sets from the literature and the Array Express database of gene expression studies (n = 21, Additional file 2: Table S2). Also, gene sets representing general processes related to PA was extracted from Molecular Signatures Database (MSigDB [20], n = 27). The general processes included inflammatory pathways, oxidation, oxidative stress, and cell cycle pathways, as well as specific molecular pathways like PTEN signaling and Toll like receptor signaling.

### Results

The mean age of women in our sample was 54.3, mean BMI was 25.3, and 25% were current smokers (Table 1). The women in the high and very high PA level groups were less likely to be smokers, had a lower BMI and were more likely to have used ATC N type medications compared to women in the low PA category. Age differed statistically significant between low and very high PA groups. However, the actual difference in number of years was small (1.4 years), thus we considered it not to be biologically relevant, and consequently we did not adjust for age (Table 2).

**Table 1:** Descriptive characteristics of the Norwegian Women and Cancer Post-genome study population (n = 871)

Characteristics	Value
Age (year), mean (SD)	54.3 (4.0)
BMI (kg/m^2^), mean (SD)	25.3 (3.9)
Smoking during past week, n (%)	
No	652 (75.0)
Yes	217 (25.0)
Medication use during past week, n (%)	
No	384 (55.0)
Yes	469 (45.0)

*BMI* body mass index, *SD* standard deviation

**Table 2:** Descriptive characteristics of the Norwegian Women and Cancer Post-genome study population according to physical activity level

	Physical activity level		p-value
	Low (3–4)	High (7–8)	
n (%)	169 (18.6)	283 (31.2)	
Age (year), mean (SD)	54.6 (4.1)	54.3 (4.1)	0.37
BMI (kg/m^2^), mean (SD)	26.3 (4.8)	24.3 (3.2)	< .0001
Smoking during past week, n (%)			0.02
No	119 (70.4)	226 (79.9)	
Yes	50 (29.6)	57 (20.1)	
ATC medication group N, n (%)			0.001
No	124 (73.4)	242 (85.5)	
Yes	45 (26.6)	41 (14.5)	
ATC medication group C, n (%)			0.07
No	132 (78.1)	240 (84.8)	
Yes	37 (21.9)	43 (15.2)	
ATC medication group M, n (%)			0.23
No	142 (84.0)	249 (88.0)	
Yes	27 (16.0)	34 (12.0)	
ATC medication group B, n (%)			0.39
No	163 (96.5)	268 (94.7)	
Yes	6 (3.5)	15 (5.3)	

	Physical activity level		p-value
	Low (3–4)	Very high (9–10)	
n (%)	169 (18.6)	50 (5.5)	
Age (years), mean (SD)	54.6 (4.1)	53.2 (3.9)	0.02
BMI (kg/m^2^), mean (SD)	26.3 (4.8)	24.0 (3.5)	< .0001
Smoking during past week, n (%)			0.74
No	119 (70.4)	34 (68.0)	
Yes	50 (29.6)	16 (32.0)	
ATC medication group N, n (%)			0.07
No	124 (73.4)	45 (84.0)	
Yes	45 (26.6)	7 (16.0)	
ATC medication group C, n (%)			0.55
No	132 (78.1)	41 (82.0)	
Yes	37 (21.9)	9 (18.0)	
ATC medication group M, n (%)			0.07
No	142 (84.0)	47 (94.0)	
Yes	27 (16.0)	3 (6.0)	
ATC medication group B, n (%)			0.19
No	163 (96.5)	46 (92.0)	
Yes	6 (3.5)	4 (8.0)	

*BMI* body mass index, *SD* standard deviation, *ATC* Anatomical Therapeutic Chemical Classification System (N: nervous system, C: cardiovascular system, M: musculo-skeletal system, B: blood and blood forming organs)

At the single-gene level, we found no associations between PA levels and gene expression profiles, neither in categorical analysis of low versus high, nor in low versus very high PA levels. Table 3 shows the top ten genes sorted by fold change. Similarly, analyzing PA levels as a continuous variable did not produce results that met our criteria for statistical significance (adjusted p < 0.05). For gene set analysis, we first employed the overall test for significance in the global test R-package. In the comparison of low versus high PA levels and low versus very high PA, the p-values were 0.82 and 0.63, respectively. As recommended by Goeman et al. when overall statistically significant level is low [19], we proceeded with a targeted approach for gene set testing. The 48 gene sets we chose were tested using the global test, but no gene sets reached statistical significance at adjusted p < 0.05.

**Table 3:** Top 10 differentially expressed genes according to log fold change between low and very high physical activity levels

Gene symbol	Gene name	Log fold change	Unadjusted p-value	FDR
RPL14	ribosomal protein L14	− 0.29	0.02	0.99
ZNF683	zinc finger protein 683	− 0.26	0.02	0.99
CLEC2D	C-type lectin domain family 2 member D	− 0.26	0.02	0.99
HBG2	hemoglobin subunit gamma 2	0.26	0.11	0.99
HBG1	hemoglobin subunit gamma 1	− 0.25	0.11	0.99
FGFBP2	fibroblast growth factor binding protein 2	− 0.24	0.02	0.99
GZMB	granzyme B	− 0.23	0.03	0.99
LOC390354	N/A	− 0.23	0.03	0.99
GZMH	granzyme H	− 0.23	0.09	0.99
RPS28	ribosomal protein S28	− 0.22	0.11	0.99

*FDR* False discovery rate

### Discussion

In the present cohort study of Norwegian, middle-aged women, we explored associations between everyday PA levels and blood gene expression profiles. Using statistical methods that are sensitive to low-magnitude associations, we found no statistically significant associations when comparing low versus high PA, low versus very high, or when using PA as a continuous variable.

Most published studies on the molecular effects of PA has focused on exercise as the exposure, in contrast to the total, everyday level of PA. Available studies often use an experimental approach by subjecting participants to exercise interventions, and include young participants, frequently males. In comparison, the present study includes participants representing the general, middle-aged female population, and provide insight into the magnitude of the effects of differing levels of total, everyday PA. Our findings are more relevant to the general population, and may serve to moderate results from controlled trials of exercise.

In addition to the single gene level analysis limma, we chose the global test when analyzing gene sets. The global test method used herein is found to be among the most sensitive methods, and not to be prone to false positive findings [21–23].

A main strength of this study is the large study population, as compared to the available literature on molecular mechanisms of PA. Smaller studies are prone to selection bias and reduced generalizability. Our large sample size gives the opportunity to discuss associations in the general population, with increased generalizability of the findings as compared to smaller studies. Further, the full-genome expression analysis method gives a global view of the actively transcribed genes in the entire blood cell pool, as opposed to studying single genes and single cell types. Finally, a validation study using objective measures showed that the global PA scale is able to rank study participants from very low to high PA levels, although the dose is not possible to determine [24].

In our analysis of healthy, middle-aged Norwegian women, self-reported PA was not associated with differences in blood gene expression profiles. To our knowledge, this is the first study assessing the association between blood gene expression profiles and everyday PA levels in a general, female population. When comparing studies of exercise to our study of overall everyday PA levels, the lack of knowledge on potential threshold levels of PA for immunological effects becomes evident. Thus, more research is needed to identify the level of PA needed for positive immunological effects, and whether these levels differ between population strata. To identify this, PA needs to be assessed using objective measures.

## Limitations

A limitation of our work is that in the observational study design, we cannot exclude the possibility of residual confounding by diet or other lifestyle factors, which may influence our results. Furthermore, we did not have available data on blood cell subpopulation distributions, which may be considered a potential confounding factor. Our sample size was relatively large compared to other published studies, however, it is possible that some of the analyses were underpowered, as we confined our analytical sample only to women that were within tested PA categories. Even though the PA scale used was shown to be reliable in correctly separating women based on their PA level, this does not exclude the possibility that the PA levels were overestimated across the PA categories used in the analyses. However, we do not expect that this overestimation differs along the scale. Further, the PA scale used captures total PA levels, including occupational PA. It was previously shown that occupational PA was associated with a lower level of perceived health [25]. This indicates that occupational PA might have unfavorable effect on anti-inflammatory gene expression, in other words, it might counteract the favorable effects of non-occupational PA. Finally, there was some time gap between questionnaires and blood sampling, so our results will only reflect potential long-term effects of overall, habitual PA levels. Taken together, our exposure measurement may not be very precise, driving our results toward the null due to potential misclassification.

## Supplementary information

**Additional file 1: Table S1.** Translation of the question on physical activity, as it appears in the NOWAC questionnaires.

**Additional file 2: Table S2.** Gene expression studies used as input for gene set analyses.

## Data Availability

The dataset used will be made available upon request. Please contact the corresponding author.

## Abbreviations

ATC: Anatomical Therapeutic Chemical Classification
BMI: Body mass index
FDR: False discovery rate
MSigDB: Molecular Signatures Database
NOWAC: Norwegian Women and Cancer
PA: Physical activity
