# Supplementary material for: Physical activity and blood gene expression profiles: the Norwegian Women and Cancer (NOWAC) Post-genome cohort
Source: BMC Res Notes. 2020 Jun 11;13:283. doi: 10.1186/s13104-020-05121-2 (PMC7291748; doi:10.1186/s13104-020-05121-2)
Supplement: Supplementary file 2 — Additional file 2: Table S2. Gene expression studies used as input for gene set analyses. [file 13104_2020_5121_MOESM2_ESM.docx]

Table S2 Gene expression studies used as input for gene set analyses

| **Author, year** | **Exposure** | **Duration of exposure** | **Study population** | **Study n** | **n gene sets*** | **Ref.** |
| --- | --- | --- | --- | --- | --- | --- |
| Buttner, 2007 | Treadmill | Acute bout | Male students | 5 | 1 | (1) |
| Carlson, 2011 | Squats and leg press | Acute bout | Young, adult males | 10 | 2 | (2) |
| Connolly, 2014 | Ergometer cycling | Acute bout | Healthy, adult males | 15 | 1 | (3) |
| Dias, 2015 | Exercise training | 18 weeks | Healthy, adult males | 13 | 4 | (4) |
| Kimsa, 2014 | Ergometer cycling | Acute bout | Male cyclist | 3 | 1 | (5) |
| Mukherjee, 2014 | Endurance exercise | Acute bout | Middle-aged male masters athletes | 17 | 4 | (6) |
| Radom-Aizik, 2009^1^ | Ergometer cycling | Acute bout | Early and late pubertal females | 20 | 1 | (7) |
| Radom-Aizik, 2009^2^ | Ergometer cycling | Acute bout | Early and late pubertal males | 20 | 1 | (8) |
| Radom-Aizik, 2014 | Ergometer cycling | Acute bout | Healthy males | 12 | 2 | (9) |
| Sakharov, 2012 | Treadmill | Acute bout | Male skiers | 19 | 2 | (10) |
| Thompson, 2010 | Exercise training | 24 weeks | Middle-aged men | 41 | 1 | (11) |
| Xiang, 2014 | Marathon | Acute bout | Recreational runners, both sexes | 16 | 1 | (12) |

*) Number of gene sets extracted from the publication

**Supplementary references**

1. Buttner P, Mosig S, Lechtermann A, Funke H, Mooren FC. Exercise affects the gene expression profiles of human white blood cells. J Appl Physiol (1985). 2007;102(1):26-36.

2. Carlson LA, Tighe SW, Kenefick RW, Dragon J, Westcott NW, Leclair RJ. Changes in transcriptional output of human peripheral blood mononuclear cells following resistance exercise. European journal of applied physiology. 2011;111(12):2919-29.

3. Connolly PH, Caiozzo VJ, Zaldivar F, Nemet D, Larson J, Hung SP, et al. Effects of exercise on gene expression in human peripheral blood mononuclear cells. J Appl Physiol (1985). 2004;97(4):1461-9.

4. Dias RG, Silva MS, Duarte NE, Bolani W, Alves CR, Junior JR, et al. PBMCs express a transcriptome signature predictor of oxygen uptake responsiveness to endurance exercise training in men. Physiol Genomics. 2015;47(2):13-23.

5. Kimsa MC, Strzalka-Mrozik B, Kimsa MW, Gola J, Kochanska-Dziurowicz A, Zebrowska A, et al. Differential expression of inflammation-related genes after intense exercise. Prague Med Rep. 2014;115(1-2):24-32.

6. Mukherjee K, Edgett BA, Burrows HW, Castro C, Griffin JL, Schwertani AG, et al. Whole blood transcriptomics and urinary metabolomics to define adaptive biochemical pathways of high-intensity exercise in 50-60 year old masters athletes. PLoS One. 2014;9(3):e92031.

7. Radom-Aizik S, Zaldivar F, Jr., Leu SY, Cooper DM. A brief bout of exercise alters gene expression and distinct gene pathways in peripheral blood mononuclear cells of early- and late-pubertal females. J Appl Physiol (1985). 2009;107(1):168-75.

8. Radom-Aizik S, Zaldivar F, Jr., Leu SY, Cooper DM. Brief bout of exercise alters gene expression in peripheral blood mononuclear cells of early- and late-pubertal males. Pediatr Res. 2009;65(4):447-52.

9. Radom-Aizik S, Zaldivar FP, Jr., Haddad F, Cooper DM. Impact of brief exercise on circulating monocyte gene and microRNA expression: implications for atherosclerotic vascular disease. Brain Behav Immun. 2014;39:121-9.

10. Sakharov DA, Maltseva DV, Riabenko EA, Shkurnikov MU, Northoff H, Tonevitsky AG, et al. Passing the anaerobic threshold is associated with substantial changes in the gene expression profile in white blood cells. European journal of applied physiology. 2012;112(3):963-72.

11. Thompson D, Markovitch D, Betts JA, Mazzatti D, Turner J, Tyrrell RM. Time course of changes in inflammatory markers during a 6-mo exercise intervention in sedentary middle-aged men: a randomized-controlled trial. J Appl Physiol (1985). 2010;108(4):769-79.

12. Xiang L, Rehm KE, Marshall GD, Jr. Effects of strenuous exercise on Th1/Th2 gene expression from human peripheral blood mononuclear cells of marathon participants. Molecular immunology. 2014;60(2):129-34.
