# Supplementary material for: Physical activity and blood gene expression profiles: the Norwegian Women and Cancer (NOWAC) Post-genome cohort
Source: BMC Res Notes. 2020 Jun 11;13:283. doi: 10.1186/s13104-020-05121-2 (PMC7291748; doi:10.1186/s13104-020-05121-2)
Supplement: Supplementary file 1 — Additional file 1: Table S1. Translation of the question on physical activity, as it appears in the NOWAC questionnaires. [file 13104_2020_5121_MOESM1_ESM.pdf]

Physical activity

Please indicate the level of your physical activity on a scale from very low to very high (at the ages of 14 and 30 years, and today). The scale below goes from 1-10. By physical activity we mean both work in and outside the home, as well as training/exercise and other physical activity, such as walking, etc. Mark the number that best describes your level of physical activity

Age	Very low									Very high
At 14 years	1	2	3	4	5	6	7	8	9	10
At 30 years	1	2	3	4	5	6	7	8	9	10
Today	1	2	3	4	5	6	7	8	9	10
